# Soil Water Content Prediction Using Electrical Resistivity Tomography (ERT) in Mediterranean Tree Orchard Soils

**DOI:** 10.3390/s22041365

**Published:** 2022-02-10

**Authors:** José A. Acosta, María Gabarrón, Marcos Martínez-Segura, Silvia Martínez-Martínez, Ángel Faz, Alejandro Pérez-Pastor, María Dolores Gómez-López, Raúl Zornoza

**Affiliations:** 1 Departamento de Ingeniería Agronómica, Universidad Politécnica de Cartagena, Paseo Alfonso XIII, 48, 30230 Cartagena, Spain; maria.gabarron@upct.es (M.G.); silvia.martinez@upct.es (S.M.-M.); angel.fazcano@upct.es (Á.F.); alex.perez-pastor@upct.es (A.P.-P.); lola.gomez@upct.es (M.D.G.-L.); raul.zornoza@upct.es (R.Z.); 2 Departamento de Ingeniería Minera y Civil, Universidad Politécnica de Cartagena, Paseo Alfonso XIII, 52, 30230 Cartagena, Spain; marcos.martinez@upct.es

**Keywords:** electrical resistivity, tomography, regulated deficit irrigation, soil moisture, nonlinear regression analysis

## Abstract

Water scarcity in arid and semiarid regions poses problems for agricultural systems, awakening special interest in the development of deficit irrigation strategies to improve water conservation. Toward this purpose, farmers and technicians must monitor soil water and soluble nutrient contents in real time using simple, rapid and economical techniques through time and space. Thus, this study aimed to achieve the following: (i) create a model that predicts water and soluble nutrient contents in soil profiles using electrical resistivity tomography (ERT); and (ii) apply the model to different woody crops under different irrigation regimes (full irrigation and regulated deficit irrigation (RDI)) to assess the efficiency of the model. Simple nonlinear regression analysis was carried out on water content and on different ion contents using electrical resistivity data as the dependent variable. A predictive model for soil water content was calibrated and validated with the datasets based on exponential decay of a three-parameter equation. Nonetheless, no accurate model was achieved to predict any soluble nutrient. Electrical resistivity images were replaced by soil water images after application of the predictive model for all studied crops. They showed that under RDI situations, soil profiles became drier at depth while plant roots seemed to uptake more water, contributing to reductions in soil water content by the creation of desiccation bulbs. Therefore, the use of ERT combined with application of the validated predictive model could be a sustainable strategy to monitor soil water evolution in soil profiles under irrigated fields, facilitating land irrigation management.

## 1. Introduction

More than one hundred countries in the world are under conditions of aridity and semi-aridity. Africa is the continent most damaged by desertification followed by Asia, Latin America and the Caribbean. On the European continent, Mediterranean countries that comprise Spain, Portugal, Italy, Turkey and Greece make up one of the four zones determined by the UN convention as being affected by desertification [1]. Hence, water scarcity in arid and semiarid regions poses widespread problems for agricultural systems, awakening special interest in the development of efficient irrigation strategies that facilitate water saving [2]. The adoption of new strategies to optimize irrigation by reducing water and energy consumption will be essential to maintain agricultural activity in desertification-affected regions. With regard to this, the Segura basin (SE Spain) has a water structural deficit of about 460 hm^3^ per year, making it the region with the greatest water deficit in the European Union; it is also the most regulated basin in Europe with the highest water use efficiency [3]. Despite its water scarcity, productive agriculture is developed there whose production is mainly exported to EU state members. Hence, irrigation of the Segura basin leads in demand for the proportion of water it needs.

Currently, special interest has arisen for the development of regulated deficit irrigation (RDI) strategies that strive to significantly reduce irrigation water content without affecting production or crop quality [4,5,6]. RDI is based on reduction of water supply during non-critical periods, covering water needs during critical periods and maximizing at the same time production per unit of applied water [7]. Nonetheless, its success greatly depends on adequate application of the water deficit. RDI also requires continuous and precise control of the status of plants and soils in order to adjust water supplies at every crop phenological period [8]. Consequently, there is a need to develop rapid, economical and easy-to-use tools so that farmers and technicians can continuously monitor soil water content in real time using soil profiles.

Methods for soil moisture determination are usually based on direct measures which consist of weighing soil before and after oven-drying, or using in situ probes. Recent studies have demonstrated the potential use of some geophysical techniques, such as electrical resistivity tomography (ERT), for soil characterization and particularly for soil moisture estimation [9,10,11,12]. While traditional techniques seem restricted to the rhizosphere area and provide data from a single point of measure, the ERT technique offers continuous measurements at multiple lengths and depths. The ERT technique is rapid, cost-effective and does not cause strong perturbations in soils [13]. This approach is based on the relationship between the behavior of a soil property and a real resistivity measure using regression models. Polynomial functions or power functions are usually used for a large range of moisture changes, typically from full saturation to dry soil states [14,15]. However, in some particular cases, this relationship can follow a linear model, as reported by Michot et al. [11]. Thus, these results highlight the large variability of data and the complexity in developing predictive models using ERT.

The ERT technique is based on the use of electric current to assess electrical properties of the subsurface (e.g., resistivity, conductivity). A set of four electrodes driven into the soil injects currents (two electrodes) and measures potential differences (two electrodes) simultaneously. The interactions of these currents with subsurface materials act differently depending on characteristics of the subsurface, allowing geoelectric profiles to be calculated [16]. Thus, soil electrical resistivity mainly involves constant physical properties of the soil, such as clay content, but it also involves variable properties such as soil water content, soil water electrical conductivity and temperature [11,17].

Electrical profiling using ERT requires many electrodes connected to a switch box by a cable. This allows multiple apparent resistivity measurements to be made using different electrodes. This system is called a multi-electrode system, or sometimes a multi-channel system [18]. Subsequently, resistivity values are obtained from the inversions of apparent resistivity values measured in the field [19,20].

Based on the aforementioned approach, we hypothesized that the ERT method could replace moisture probes in woody crops during the vegetation period using a moisture-resistivity model. Thus, an experiment was set up in different commercial tree orchards where full irrigation and RDI were applied over a period of two years. The objectives of the experiment were the following: (i) to calibrate a model that allows users to predict water and soluble nutrient contents in soil profiles of different woody crops using ERT; (ii) to validate the model in woody crop orchards under different irrigation regimes in order to assess the efficiency of the model; and (iii) to assess differences in soil water content using soil profiles from different irrigation schedules in different crops. We hypothesized that ρ values derived from ERT could be related to soil moisture and some nutrient contents by the use of nonlinear regression analysis, thereby calibrating and validating a suitable model. The application of this model would permit users to depict soil profile images with estimated values of soil, water and nutrient concentrations for rapid and cost-efficient monitoring of these properties by end-users.

## 2. Materials and Methods

### 2.1. Study Area

**Figure 1:**
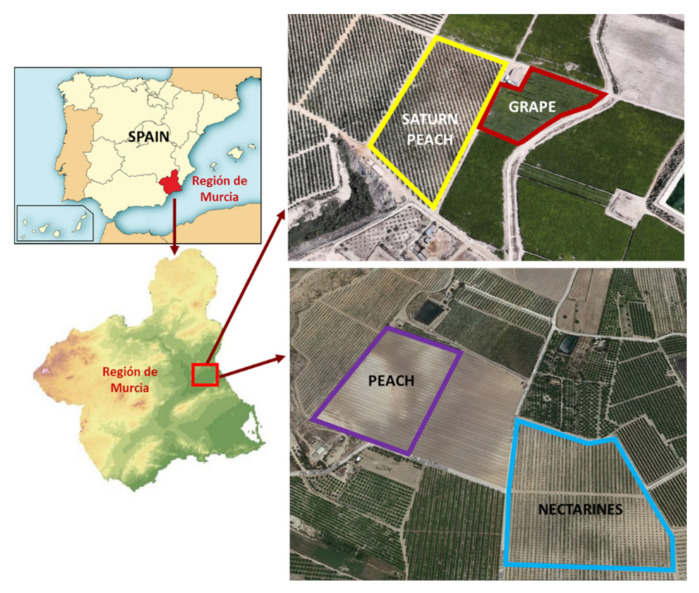
Locations of the selected orchards.

The study was conducted at three commercial farms located in Campotéjar (Region of Murcia, Spain) where different crops were selected. The first farm (38°11′ N; 1°18′ W) had Saturn peach (*Prunus persica* var. *platycarpa*) and table grape (*Vitis vinifera*) crops. The *P. persica* var. *platycarpa* crop had an extension of 4752 m^2^, with 288 trees at spacings of 5.5 m between rows and 3.5 m between trees within the same row; the *V. vinifera* crop had an extension of 3520 m^2^, with 252 trees at spacings of 3.5 m between rows and 3.0 m between trees within the same row. The second farm (38°17′ N; 1°23′ W) had peach trees (*Prunus persica*), with an extension of 4725 m^2^, with 270 trees at spacings of 5.5 m between rows and 3.5 m between trees within the same row. The third farm (38°13′ N; 1°22′ W) was cultivated with nectarine trees (*Prunus persica* var. *nucipersica*), with an extension of 670,000 m^2^, with 31,900 trees at spacings of 6 m between rows and 3.5 m between trees within the same row (Figure 1).

The climate of the area is semiarid Mediterranean with a mean annual temperature of 18 °C and a mean annual precipitation of 270 mm. Potential evapotranspiration surpasses 1200 mm per year. The geology of the area is represented by Neogene and Quaternary marls [21] and soils are classified as Calcaric Regosols [22] with soil depths of Ap:0–45 cm and C:>45 cm for Saturn peach; Ap:0–35 cm and C:>35 cm for grape; Ap:0–40 cm and C:>40 cm for peach; and Ap:0–50 cm and C:>50 cm for nectarine. The main soil characteristics for all crops are shown in Table 1. Customary cultural practices (e.g., weed control, fertilization, pruning, fruit thinning and banding) are carried out by the technical departments of these commercial orchards.

A drip irrigation system was installed, with two lines per tree row and nine pressure-compensated emitters (1.6 L h^−1^) per tree placed at every 75 cm. Irrigation was scheduled weekly at nights. The frequency of irrigation varied according to evaporative demand, which was 1 to 2 times per week in winter, 2 to 7 times per week in spring and autumn, and 7 to 14 times per week in summer. All crops were irrigated with well water having an electrical conductivity ranging between 2.5 and 2.8 dS m^−1^. We applied two different irrigation treatments as follows: (1) a control group (CT) where trees were irrigated to satisfy the maximum water requirement of each crop type; and (2) a regulated deficit irrigation (RDI) group where trees were irrigated at 100% of the crop water requirement except for the post-harvest period in *P. persica* var. *platycarpa*, the post-veraison period in *V. vinifera* and the pre-harvest and post-harvest periods in *P. persica* var. *nucipersica*, which were irrigated at 50% CT; the aim of this was not to surpass the threshold of the stem water potential of −2 MPa. The total crop water needs were estimated as the product of reference crop evapotranspiration (ET0) and the crop coefficients (between 0.25 and 0.55) proposed by the Agricultural Information System of Murcia (http://siam.imida.es, 1 March 2015) for this area, adjusted for tree size [23]. The experiment was set as a randomized design with three replications per treatment. Each replicate had three adjacent tree rows and fifteen trees per row. All measurements and samplings were carried out in the central row of each replicate.

### 2.2. ERT Methodology

**Figure 2:**
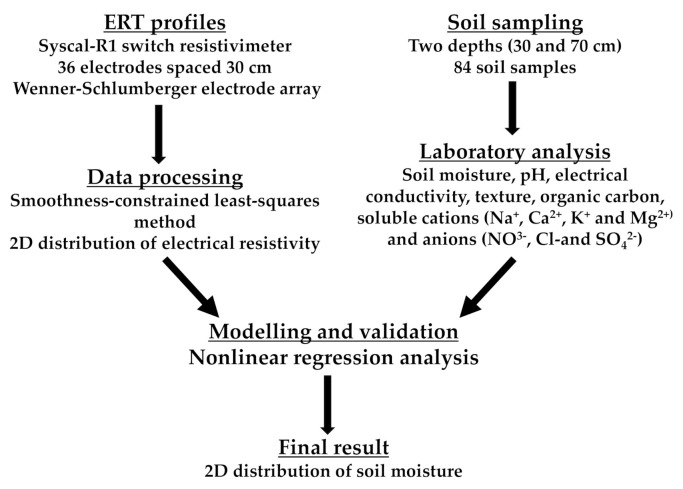
Scheme of the methodology applied in our study.

The combination of electrical sounding and profiling methods in a single process (2D resistivity imaging) allows the ERT method to provide information about lateral and vertical resistivity changes along a soil profile [24,25]. Apparent resistivity measurements were obtained using a computer-controlled multielectrode system consisting of a Syscal-R1 switch resistivimeter (IRIS Instruments, 2001) connected to 36 stainless steel electrodes spaced 30 cm from each other. The electrodes were georeferenced using a GPS unit, allowing characterization of the soil to a depth of 2 m (Figure 2). Connections between the resistivimeter and electrodes were made using a multicore cable and takeout clips for galvanic coupling of the electrodes to the ground. The datasets were acquired using a Wenner–Schlumberger electrode array due to its high signal-to-noise ratio and good vertical resolution [21].

Campaigns of ERT were carried out during the summer periods of 2015 and 2016 (July–August) at the previously mentioned commercial farms. The summer period was selected since deficit irrigation was applied to all crops at this time, and the differences between CT and RDI treatments were at their greatest. This is important since electrical resistivities of soils depend on amounts of water and dissolved ions present in the pores [26]. The ERT campaigns were performed in one of the irrigation lines along the 15 trees in the central row of each plot.

**Figure 3:**
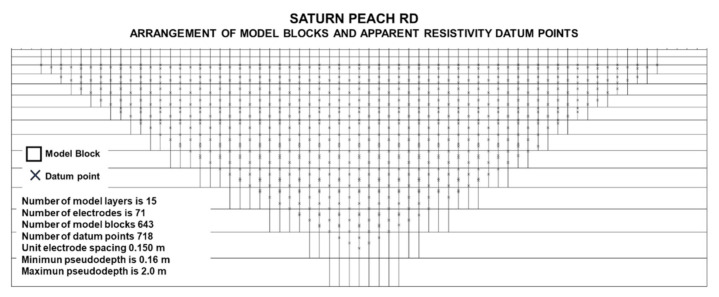
Arrangement of electrodes for a 2D electrical survey and pseudosection data patterns for the Wenner–Schlumberger arrays.

Data obtained were analyzed with all erroneous values first removed before inversion in the PROSYS software. Data were subsequently processed by the RES2DINV software, which runs an inversion process based upon the smoothness-constrained least-squares method in order to obtain a 2D distribution of electrical resistivity. Thus, the 2D distribution is related to physical properties of the subsurface named as an inverted resistivity image or 2D resistivity section [27]. The accuracy of the inversion model of each section was characterized by its root-mean-square (RMS) error value [13,28]. The inversion software (RES2DINV) divided the subsurface into rectangular pixels with each of them taking a singular resistivity value according to the material present in it. ERT resolution depends on electrode spacing and resistivity contrast; vertical resolution decreases as depth increases [29]. In our study, the pixel width was 0.15 m and the vertical pixel dimension was around 0.16 m. Pixel shape achieved in this study was sufficient to distinguish property changes at shallow depths [30]. Figure 3 shows data numbers (700 data points) obtained in one electrical tomography profile.

### 2.3. Soil Sampling and Analytical Methods

Immediately after the ERT campaigns, soil samples were collected along the ERT profiles at different electrode positions at two different depths (0–30 cm and 0–70 cm). A total of 84 soil samples were collected, 12 for each crop type and year. Soil samples were collected with an auger hole. For each depth, soil was introduced into a polyethylene bag and mixed, after which samples were transported to the laboratory where oven-drying was applied for 48 h at 45 °C. Samples were passed through a 2-millimetre sieve for analyses.

Soil moisture was measured in situ using a ProCheck and 5TM sensors (Decagon Devices, Pullman, WA, USA). Soil pH and electrical conductivity (EC) were measured in deionised water (1:1 and 1:5 *w*/*v*, respectively). Soil texture was measured from a soil/Na-polyphosphate extract and determined using laser diffraction (Mastersizer 2000, Malvern Panalytical, Malvern, UK). Soil organic carbon (SOC) was determined using the dichromate oxidation method [31], CaCO_3_ was determined using the Bernard’s calcimeter and total nitrogen (NT) was determined using the Kjeldahl method [32]. Soluble cations (Na^+^, Ca^2+^, K^+^ and Mg^2+^) and anions (NO_3_^−^, Cl^−^ and SO_4_^2−^) were extracted with deionized water (1:5 *w*/*v*). Anions were measured using ion chromatography (ICNet, Metrohm, Herisau, Switzerland), while cations were measured using atomic absorption spectrometry (AAnalyst 800, Perkin Elmer, Waltham, MA, USA).

### 2.4. Data Analyses

A Kolmogorov–Smirnov normality test at *p* < 0.05 was used to ensure normality in fitting the data. No normal distribution of data was achieved even after log-transformation. Consequently, a Mann–Whitney U test at *p* < 0.05 was performed in order to assess significant differences among CT and RDI treatments for each variable. A Spearman correlation was carried out to establish relationships between electrical resistivity data and soil physicochemical properties. These statistical analyses were performed with the software IBM SPSS statistics v.23.

Some studies assess the relationship between moisture and resistivity applying Archie’s laws; however, this is not recommended for soils with sand and coarse-sized materials content below 80% [9] or for heterogeneous soils [13,15]. Therefore, in our study a simple nonlinear regression analysis was carried out in order to obtain a model that allowed prediction of soil water content and soluble nutrients in crop fields based on electrical resistivity data. The selected response variable (Y) was the soil water content or the contents of different cations and anions, while the soil resistivity or log-resistivity was used as the explanatory variable for prediction (X). Of the total data, 67% were used as the calibration set, while the remaining 33% of the total data were used as the validation set. Outlier data were excluded from the analyses. Thus, 53 values were used for calibration and 31 values used for validation. Residuals from the calibrated models were satisfactory checked for the model assumptions of normality (Kolmogorov–Smirnov test), linearity and homocedasticity, and equations with R^2^ < 0.6 were rejected. In order to validate the models, residuals from the estimated variables of the validation set had to be within the confidence interval (CI) (at 95%) of the residual distribution of the calibrated model. CI was calculated as ±1.96 standard deviations of the residuals in the calibrated model. Modelling was performed with the software SigmaPlot v.12 (Systat Software, Inc., San Jose, CA, USA).

## 3. Results and Discussion

### 3.1. ERT Model

We observed that log-resistivity was negatively and significantly correlated with soil moisture (R = −0.545; *p* < 0.05), EC (R = −0.305; *p* < 0.01), NO_3_^−^ (R = −0.295; *p* < 0.01), SO_4_^2−^ (R = −0.380; *p* < 0.05) and Mg^2+^ (R = −0.378; *p*-value < 0.05). The negative correlation suggests that a high concentration of water and salts is followed by low values of electrical resistivity [14]. Nonetheless, correlation coefficients were low, indicating a lack of any strong linear correlations between electrical resistivity and other variables.

**Figure 4:**
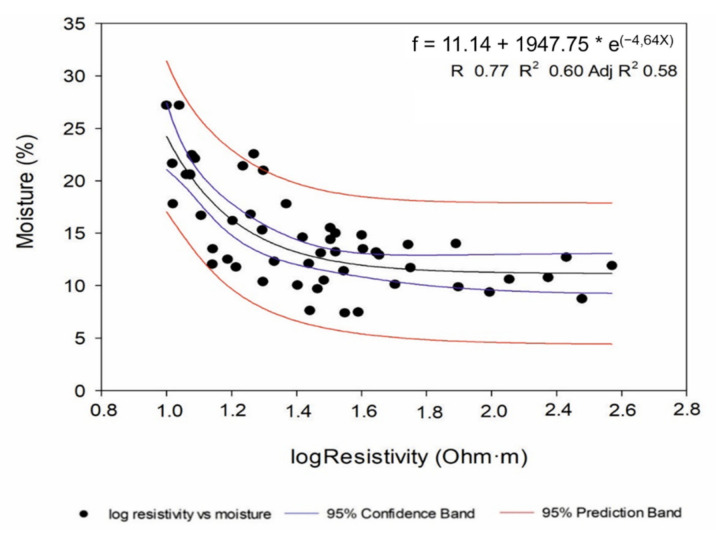
Calibrated model (exponential decay of a three-parameter equation) for soil moisture estimation using the logarithm of electrical resistivity as the explanatory variable (*n* = 53).

As a result of the simple nonlinear regression, multiple equations were obtained for each dependent variable. However, among all of them, only an exponential decay of a three-parameter equation between soil moisture and log-resistivity achieved all assumptions for model acceptance (residuals normality (Z = 0.54; *p* < 0.05), linearity and homoscedasticity, and R^2^ ≥ 0.6) (Figure 4). The lack of any strong correlations between electrical resistivity and the different cations and anions hampered the calibration of suitable models. In this sense, soil moisture was the only variable that showed a strong correlation (R > 0.5) with resistivity. Hadzick et al. [33] were also able to calibrate ERT data with soil water content using regression equations, although in linear dependence. These authors reported that the accuracy of the regression model increased with increasing soil depth, showing values of R^2^ = 0.4–0.6 for 30 cm and 70 cm soil depths, similar to those obtained in this study for the exponential equation. Farzamian et al. [10] obtained moisture distribution maps from exponential equations obtained in a regression analysis comparing degree of saturation versus resistivity in unsaturated soils.

**Figure 5:**
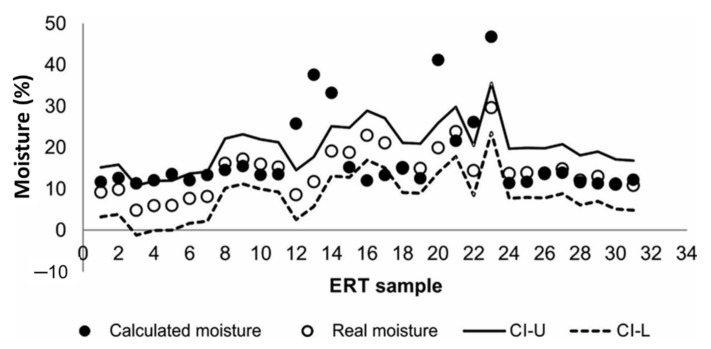
Relationship between real moisture and calculated moisture using the calibrated model (CI-U: upper limit of the confidence interval; CI-L: lower limit of the confidence level).

The efficiency of the only model obtained by nonlinear regression analysis was validated by applying the exponential decay of a three-parameter equation to the validation set. This validation was shown by plotting the actual moisture values measured in farm soils and the moisture estimated with the model using the 31 validation samples (Figure 5). A total of 65% of the estimated data were within the CI (at 95%), verifying good quality of the model. This methodology was similar to that used by other authors [9,15,29] who also faced estimated and real moisture data in plots.

### 3.2. Electrical Resistivity and Soil Moisture Imaging

Resistivities are specific to each soil type, since they depend on several soil properties such as porosity, cation exchange capacity, organic matter content, salinity or clay content [10,13,34]. In order to ensure correct application of the model to all of the studied orchards, different soils were previously studied to select those orchards that reported similar soil properties. In order to avoid clay interferences with resistivity measurements, selected soils had to exhibit low clay contents, as can be seen in Table 1.

**Figure 6:**
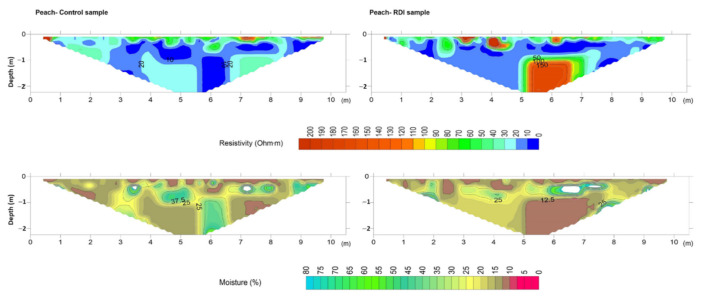
Resistivity model (**top**) and predicted soil moisture (**bottom**) for control (**left**) and deficit irrigation (**right**) in the peach orchard.

**Figure 7:**
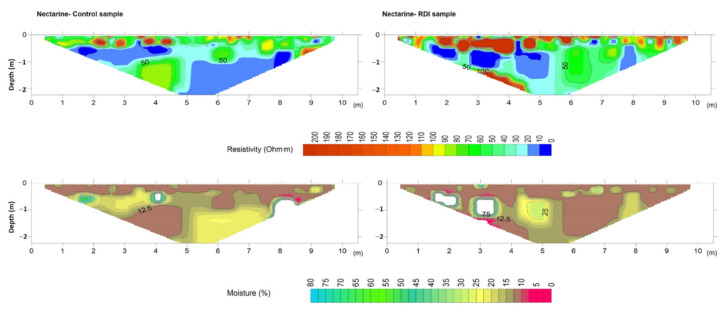
Resistivity model (**top**) and predicted soil moisture (**bottom**) for control (**left**) and deficit irrigation (**right**) in the nectarine orchard.

**Figure 8:**
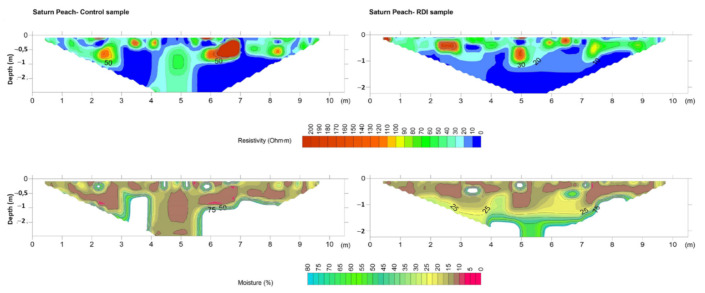
Resistivity model (**top**) and predicted soil moisture (**bottom**) for control (**left**) and deficit irrigation (**right**) in the Saturn peach orchard.

**Figure 9:**
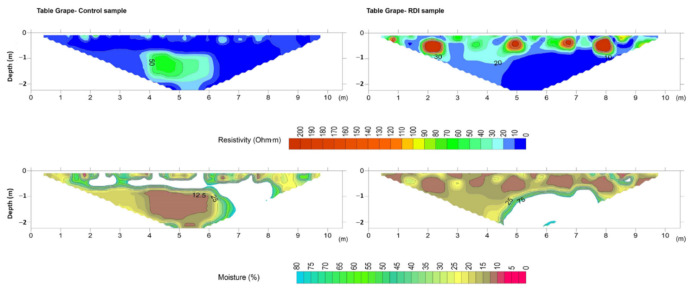
Resistivity model (**top**) and predicted soil moisture (**bottom**) for control (**left**) and deficit irrigation (**right**) in the table grape orchard.

Inversion of measured resistivities is an essential step before interpretation of ERT profiles since apparent resistivity values rarely reveal the true structure of soils [15]. As a consequence, a 2D inversion was performed in this study using a cell-based model where the subsurface is subdivided into rectangular cells. The positions of the cells are fixed and only the resistivities of cells are allowed to vary during the inversion process, being the model parameter of the resistivity for each cell [20]. Hence, after a certain number of iterations, values of the interpreted resistivities and depths were obtained and plotted using the resistivity model (Figure 6, Figure 7, Figure 8 and Figure 9).

The Mann–Whitney U test showed that there were significant differences (*p* < 0.05) between CT and RDI treatments for soil moisture, Na^+^ and Mg^2+^. However, the 2D resistivity sections were only replaced by 2D soil moisture sections by application of the validated model (Figure 4) to all electrical resistivity values obtained with each ERT campaign (Figure 6, Figure 7, Figure 8 and Figure 9). Note that white-coloured areas in the plots were considered outliers of the model developed since these data exceeded the calibration range of the model.

The expected relationship of resistivity and soil moisture is inverse, since reduction of the liquid phase decreases charge mobility (anions and electrons) [35] and should be strong enough to show variations in resistivity on the order of 10–100 Ω·m between dry and moist soil [14]. Muñoz-Castelblanco et al. [36] found that variations in the degree of water saturation between 20% and 100% corresponded to variations in electrical resistivity between 100 and 10 Ohm·.m from resistivity data for loess at 1 m of depth (e = 0.84 and e = 0.72) and at 3.3 m of depth. Figure 6 shows the electrical resistivity and moisture sections for CT and RDI in the peach (*P. persica*) orchard. Variations in soil resistivity between CT and RDI profiles were attributed to variations in soil moisture since soil conditions and composition were the same in both profiles, except for the irrigation scheme used. Thus, the CT profile showed moderate values of resistivity (~50–70 Ω·m) in the first 30 cm of depth along the central part of the profile (3–8 m). This resistivity was associated with low water content (8–12%), with opposite trends observed at the borders. Below 30 cm of depth, the section showed lower resistivity values (~0–10 Ω·m) with increased moisture values (40–45%). There were furthermore two spots of moderate resistivity (20–40 Ω·m) at 4 m and 7 m from the first electrode (0 m) and at depths of 1–2 m, which were associated with moistures of 25%.

The distribution of resistivity throughout the RDI profile was more heterogeneous than in the CT profile from depths of 50 cm. Resistivity reached values of 20 Ω·m with some low resistive spots that were coincident with water accumulation areas (25–50% of moisture). It is important to highlight the discordance of high resistivity (>100 Ω·m) and low moisture values (0–10%) observed for depths of 1–2 m and 6 m from the first electrode, which in the CT profile reported resistivity values next to zero. This suggests that under RDI water may be retained in soil sub-superficial layers (50–70 cm of depth), likely as a result of root action [37]. In this research, the ERT profiles were carried out very close to the trees, thus it is very likely that these anomalies resulted from the presence of roots. According to Giambastiani et al. [38], the presence of roots significantly affects ERT results; moreover, there are significant variations in resistivity ranges depending on the type of tree and on measurement conditions (wet or dry) [39,40,41].

In the nectarine (*P. persica* var. *nucipersica*) crop area the CT profile (Figure 7) revealed medium to high resistivity values (60–90 Ω·m) at 30–50 cm depth with high resistive soil pockets (>100 Ω·m) linked to low water content (~10%). In the RDI plot (Figure 7), the sub-superficial soil layer (30–50 cm depth) showed larger and deeper resistive areas (>100 Ω·m) than those of the CT profile. These resistivity values were linked to moisture values ≤ 10%. Furthermore, no percolation of water to deeper soil layers (>1 m) was observed. These observations suggest that in the CT crop where 100% of the water requirement was satisfied, a portion of the water was absorbed by trees while the rest percolated through the soil profile, reaching values of moisture close to 20%. In RDI the irrigation water was mainly consumed by tree roots in sub-superficial layers. Discordances in resistivity and moisture values recorded at the borders of profiles are normally a result of the border effect of the ERT model [20].

Peach and nectarine crop areas were located in farms with similar soil clay contents, with values of 16% and 13%, respectively; these could strongly affect resistivity values (Table 1). Despite both farms having very different salinity values between them (Table 1), 4.56 mScm^−1^ in peach and 1.34 mScm^−1^ in nectarine, the effect of electrical conductivity on resistivity values was not significant, since the correlation between EC and log-resistivity was low (R = −0.305; *p* < 0.01) and it did not achieve all assumptions for model acceptance. Therefore, resistivity values were mainly affected by moisture content, where resistivities > 60 Ω·m were associated with moisture contents of ~12%, while resistivities of 10–20 Ω·m were linked to moisture contents of 25%. Resistivity values < 10 Ω·m were associated with moisture contents of 40–45%.

Saturn peach (*Prunus persica* var. *platycarpa*) showed moderate resistivity values (20–50 Ω·m) at the borders and at the centre of the control pseudosection (Figure 8), and were linked to soil moisture values of 15–30%. Moreover, some resistive soil spots (90–100 Ω·m) were observed at depths of 0.5 m and 1 m, and were linked to moisture values < 12%. This suggests an effect in water movement by root water uptake [15]. In the RDI profile, low resistivity values (<10 Ω·m) were more homogeneously present in areas below 50 cm depth; this was associated with a water content of 30%. Moderately resistive soil was located over this area, showing soil pockets with high resistivities (up to 160 Ω·m) as a result of reduced water availability (10% moisture) for tree roots. Since the amount of irrigation water was lower in RDI treatment than in CT, percolation of water and soil moisture content were lower in RDI than in CT (Figure 8). White areas in both control and RDI plots were outliers of the model.

Table grape (*Vitis vinifera*) showed homogeneous resistivity behaviour throughout the profile with a resistivity of 10 Ω·m and moisture values close to 25–30%, except on the soil surface where some soil pockets of low to medium resistivity (20–30 Ω·m) were observed along with moisture values of 15–35% (Figure 9). Note that along 50 cm of depth, a strip of zero resistivity was observed, extending to 2 m of depth at the right zone of the white-coloured subsection. In this area the moisture values were considered outliers of the model. Additionally, a higher resistive patch was observed 4–6 m from the first electrode with a meter of thickness from the first meter of depth, reaching values of 80 Ω·m (moisture content <12%). Immediately below this strip, moisture values decreased from 90% to 12%, indicating that no infiltration took place. The RDI profile (Figure 9) showed resistive soil spots (>100 Ω·m) associated with moisture values close to 12%. This behaviour was not observed in the CT profile, suggesting an absence of water percolating and powerful water uptake by grape roots.

## 4. Conclusions

The results showed that log-resistivity was negatively and significantly correlated with soil moisture, EC, NO_3_^−^, SO_4_^2−^ and Mg^2+^; however, due to low values of their correlation coefficients (R < 0,55), only an exponential decay of the three-parameter equation between soil moisture and log-resistivity achieved all assumptions for model acceptance (residuals normality (Z = 0.54; *p* < 0.05), linearity and homoscedasticity, and R^2^ ≥ 0.6). The model obtained allowed assessment of spatial variabilities of water content between irrigation schedules, showing differences between full irrigation (CT) and regulated deficit irrigation (RDI) among the different crops (Saturn peach, table grape, peach and nectarine). In the CT nectarine crop, results revealed that a portion of the irrigation water was absorbed by trees while the rest percolated through the soil profile in contrast with RDI where the irrigation water was mainly consumed by tree roots in sub-superficial layers. The ERT model has shown that under deficit irrigation situations, Saturn peach trees seem to uptake water from more distant locations from their root systems than when under full irrigation. At the same time, vertical water movement was not registered in RDI, contributing to water savings. The ERT model applied to the table grape orchard under the RDI regimen suggested an absence of water percolating and powerful water uptake by grape roots. Therefore, the ERT technique could be a useful and efficient tool for estimating soil moisture by calibration of empirical nonlinear regression models and for depicting different moisture zones through the soil profile, thereby avoiding aggressive measurements in the field. In addition, the results obtained from the model can be used to calculate soil-water related coefficients, such as water stress coefficients.

## Figures and Tables

**Table 1:** Main soil properties for the different crops used in this study.

	Crops			
Soil Properties	Saturn Peach	Table Grape	Peach	Nectarine
Bulk density (g cm^−3^)	1.23	1.17	1.17	1.32
pH	7.90	7.76	7.79	8.02
Electrical conductivity 1:5 (mS cm^−1^)	3.42	2.43	4.56	1.34
Organic carbon (g kg^−1^)	9.1	12.8	10.1	12.8
Total nitrogen (g kg^−1^)	1.05	1.16	0.94	1.42
CaCO_3_ (%)	43	46	33	56
Clay (%)	10	15	16	13
Silt (%)	56	56	24	59
Sand (%)	34	29	60	28
